# Liver–Heart on chip models for drug safety

**DOI:** 10.1063/5.0048986

**Published:** 2021-07-14

**Authors:** Erika Ferrari, Marco Rasponi

**Affiliations:** Department of Electronics, Information and Bioengineering, Politecnico di Milano, 20133 Milano, Italy

## Abstract

Current pre-clinical models to evaluate drug safety during the drug development process (DDP) mainly rely on traditional two-dimensional cell cultures, considered too simplistic and often ineffective, or animal experimentations, which are costly, time-consuming, and not truly representative of human responses. Their clinical translation thus remains limited, eventually causing attrition and leading to high rates of failure during clinical trials. These drawbacks can be overcome by the recently developed Organs-on-Chip (OoC) technology. OoC are sophisticated *in vitro* systems capable of recapitulating pivotal architecture and functionalities of human organs. OoC are receiving increasing attention from the stakeholders of the DDP, particularly concerning drug screening and safety applications. When a drug is administered in the human body, it is metabolized by the liver and the resulting compound may cause unpredicted toxicity on off-target organs such as the heart. In this sense, several liver and heart models have been widely adopted to assess the toxicity of new or recalled drugs. Recent advances in OoC technology are making available platforms encompassing multiple organs fluidically connected to efficiently assess and predict the systemic effects of compounds. Such Multi-Organs-on-Chip (MOoC) platforms represent a disruptive solution to study drug-related effects, which results particularly useful to predict liver metabolism on off-target organs to ultimately improve drug safety testing in the pre-clinical phases of the DDP. In this review, we focus on recently developed liver and heart on chip systems for drug toxicity testing. In addition, MOoC platforms encompassing connected liver and heart tissues have been further reviewed and discussed.

## INTRODUCTION

The biopharmaceutical classification system classifies drugs based on their properties (i.e., physical, chemical) as well as pharmacokinetic (PK) and pharmacodynamic (PD) profiles that are derived from the complex processes of absorption, distribution, metabolism, and elimination (ADME).^1,2^ For what concerns drugs and xenobiotics metabolism and their excretion from the body, the main actively involved organ is the liver.^3^ In particular, drugs are adsorbed through the small intestine and delivered to the liver where they are subjected to three phases of metabolism. In phase I, the prodrug (i.e., parent drug) is converted into its active form by the enzymes of the cytochrome P450 (CYP450) of hepatocytes. This biotransformation can cause changes in drug bioavailability and related effects resulting in nontoxic, pharmacologically more toxic or more efficient compounds than the parent drug. In phase II, the drug is linked to radical groups (i.e., sulfation, methylation, and glucuronidation) to further increase its solubility. In phase III, the products generated during phase II are carried outside the hepatocytes to be excreted via the kidneys.^4,5^ Thus, figuring out the interactions that generate between liver and the administered drugs is a critical step during pre-clinical phases of the drug development process (DDP) as liver metabolism and related toxicity are among the major causes of drug failures and consequent withdrawals.^6^

Along with liver toxicity, heart toxicity is the other principal cause of drug failures and recalls during the DDP.^7,8^ Cardiotoxicity takes place when heart damages prevent proper pumping of blood due to alterations in the heartbeat kinetics.^9^ Cardiotoxicity represents one of the most significant types of drug-induced toxicity.^10^ A failure in predicting cardiotoxic effects in pre-clinical phases of the DDP results in rising risks and costs for the pharmaceutical industries. Moreover, a failure in recognizing cardiotoxic effects during human clinical trials may lead to the necessity of drug withdrawal from the market.^11,12^ Hence, it is essential to test compounds safety for drug-related structural (associated with a morphological damage)^13^ and functional (i.e., arrhythmias) cardiotoxicities. When dealing with functional cardiotoxicity, the most common cardiac side effect is the prolongation of the QT interval (i.e., conduction velocity in ventricles becomes abnormally slow)^14^ that can lead to ventricular fibrillation which ultimately causes brain injury and death.^15,16^ Unfortunately, these side effects often show up only late in clinical trials. Indeed, clinical phase failures are mostly related to safety concerns and drive a great loss of resources in terms of costs, time and human subjects.^17^ Therefore, to reduce the gap generated between pre-clinical and clinical phases, and thus reduce the impact of these failures, the predictivity of pre-clinical models should be improved.^18^ Currently, in the pre-clinical phases the mechanism of action of new compounds results not adequately predicted, drug doses are often ineffective when scaled to patients and diffusion kinetics found in *in vitro* and *in vivo* experiments varies dramatically.^19^ This relies on the fact that many *in vitro* models are two-dimensional (2D) and lack the complexity of the native cellular architectures, generating cell monolayers which are not properly functional in response to drugs and toxins.^19,20^ Moreover, these 2D models, are incapable of modeling situations where organ–organ communication is fundamental.^21^ In fact, in the human body cells are in a three-dimensional (3D) microenvironment, interacting with other cells as well as surrounding tissues by secreting soluble factors that mediate peripheral tissue–tissue crosstalk.^19^ In recent years novel *in vitro* tools capable of effectively predict human-related clinical outcomes have been investigated. Nonetheless, *in vitro* systems able to predict the complex ADME process of administrated drugs and their effects on target and off-target tissues still need to be improved.^22^ For example, many metabolites derived from hepatic metabolic processes are known to cause safety issues on the heart.^11^ In light of this, animal models currently represent the only approach to study the systemic response of an organism to new compounds, as they have the complexity of an entire living system and can efficiently model drug ADME process.^23^ Nonetheless, animal models suffer from several drawbacks including ethical concerns, high costs and poor translation of outcomes to humans due to species-related differences.^24,25^ In this scenario, to move on in the DDP, great efforts are being undertaken in developing engineered *in vitro* systems better representative of the *in vivo* human condition, to increase the efficiency in screening molecules in the early pre-clinical phases before they are admitted to clinical trials.^26,27^

In the last decade, advances in microfluidic technologies enabled the development of microphysiological engineered tissue models as advanced *in vitro* platforms suitable for drug screening applications.^28^ In particular, Organs-on-Chip (OoC) technology, built on the combination of human cells, 3D engineered microarchitectures and biomaterials, represents a reliable tool to model essential functions of human organs in *in vitro* controlled microenvironments.^29,30^ OoC models have proved unprecedented advantages over both traditional cell culture systems and animal models (i.e., cost effective, use of cells of human origin), in terms of high-throughput screening, drug discovery and toxicity testing.^31,32^ Among them, Multi-Organs-on-Chip (MOoC) platforms represent a disruptive solution to study drug-related effects at the tissue level on several organs simultaneously, leading to prediction of drug toxicity and ultimately improving drug safety issues.^33,34^ Their potential lies in the capacity to predict drugs bioavailability and mechanisms of action *in vitro*, thus providing knowledge on whether a drug is able to target the organ of interest or it causes off-target toxicity (e.g., cardiovascular side effects).^1^

In this review, we focus on the current microfluidic strategies to assess liver and cardiac toxicity *in vitro*. Liver-on-chip and heart-on-chip platforms for drug toxicity evaluation will be described and critically evaluated. In addition, the integration on liver and heart within multiorgan platforms for drug safety testing will be further reviewed and discussed.

## LIVER-ON-CHIP FOR DRUG TESTING

**FIG. 1.**
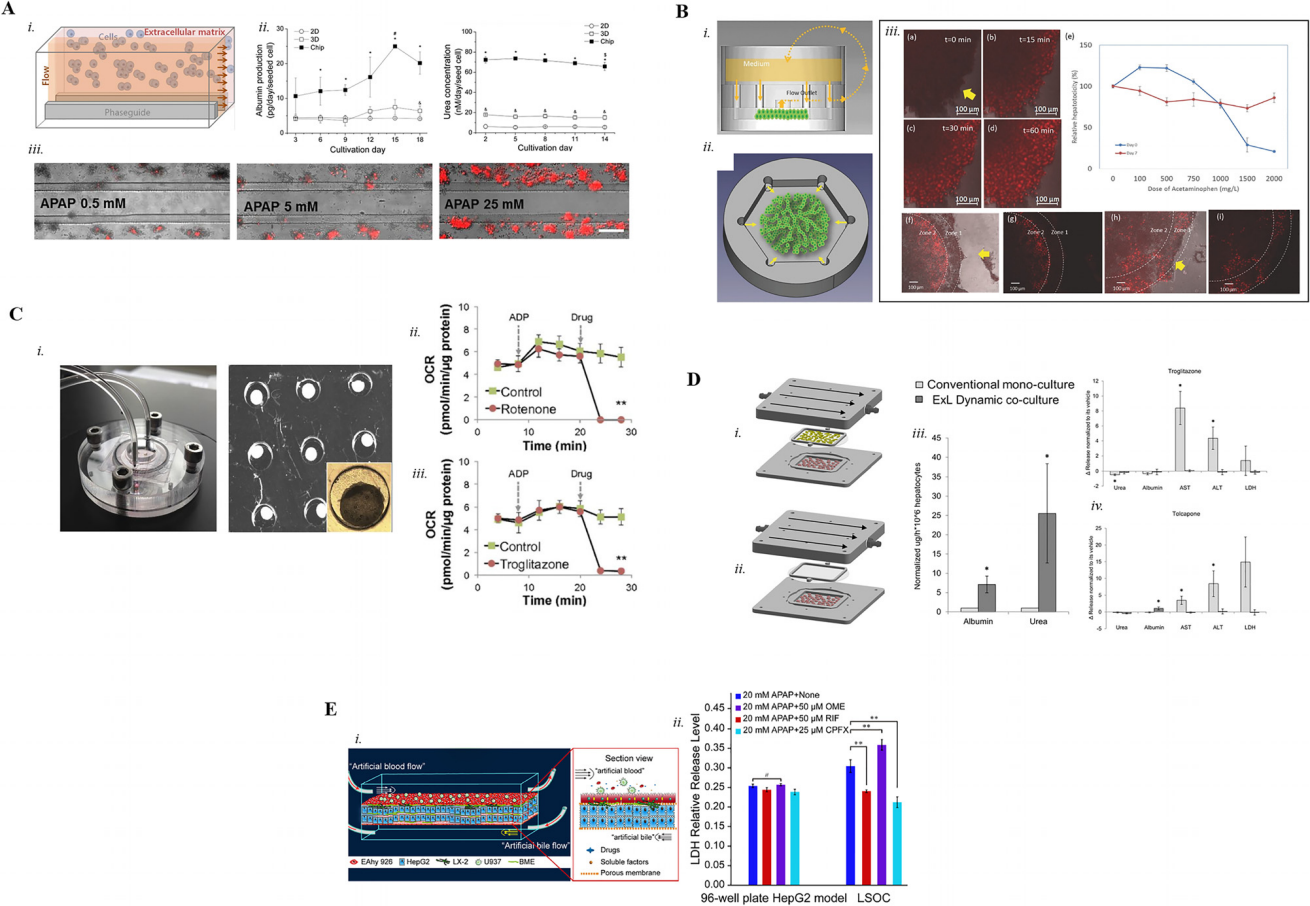
(a) 3D schematic representation of the phaseguide-based microfluidic device encompassing HepG2 cells embedded in ECM separated from fluid flow by the phaseguides (i); (ii) albumin and urea production within 18 days under 2D, 3D, and chip conditions: data are shown as mean ± SD (n = 3); (iii) phase-contrast images of HepG2 cells cultured in the perfused chip and exposed to increasing concentrations (0.5, 5, 25 mM) of APAP. Reproduced with the permission from Jang *et al.*, Biomicrofluidics **9**, 1–12 (2015). Copyright 2015 AIP Publishing.^43^ (b) Concept of the tissue incubator (i) and the schematic of the radial flow generated (ii); (iii) radial gradient and dose‐associated zonal hepatotoxicity within the device: (a)–(d) radial gradient, (e) relative APAP‐induced hepatotoxicity at day 0 and day 7 standard Petri dish, (f) and (g) recapitulation of APAP‐induced hepatotoxicity (1200 mg/l APAP) at day 7 within the device compared to day 1 (h) and (i). The yellow arrow shows the direction of the flow. Reproduced with permission from Weng *et al.*, Adv. Mater. **29**, 1–7 (2017). Copyright 2017 John Wiley and Sons.^45^ (c) Picture of assembled device and real image of HepG2/C3A organoid after overnight incubation (i); the graphs show Oxygen Consumption Rates (OCR) measured on isolated mitochondria for 30 min, followed by a first Adenosine diphosphate (ADP) injection (arrow) and a second injection of 50 *μ*M rotenone (ii) or troglitazone (iii). **P <0.01 by student's t test. Reproduced with permission from Bavli *et al.*, Proc. Natl. Acad. Sci. **113**(16), E2231–E2240 (2016). Copyright 2016 PNAS.^47^ (d) Concept of dynamic co-culture (i) optimal condition with hepatocytes (red) and liver sinusoidal endothelial cells (LSEC, yellow) and dynamic monoculture (ii); (iii) albumin production after 3 days of culture in both conditions using the Exoliver (ExL). N = 4 independent experiments and expressed as mean ± standard error of the mean. *p value <0.05 vs conventional culture; (iv) ExL‐cultured hepatocytes reaction to acute DILI (24 h with 100 *μ*M troglitazone and 100 *μ*M tolcapone): hepatocytes viability, urea and albumin synthesis, transaminases (ALT: alanine aminotransferase, AST: aspartate aminotransferase), and lactate dehydrogenase (LDH) production. N = 4 independent experiments expressed as mean ± standard error of the mean. *p value <0.05 vs its corresponding vehicle. Reproduced with permission from Ortega-Ribera *et al.*, Biotechnol. Bioeng. **115**(10), 2585–2594 (2018). Copyright 2018 Authors, licensed under a Creative Commons Attribution (CC BY) license.^54^ (e) Concept of liver sinusoid structure within the device (i); (ii) comparison between the 96-well plate HepG2 model and LoC device of drug interactions: hepatotoxicity (LDH levels) of 20 mM APAP in combination with 50 *μ*M RIF, 50 *μ*M OME, and 25 *μ*M CPFX. N = 3 experimental models. Reproduced with the permission from Deng *et al.* Biomicrofluidics. **13**(2), 024101 (2019). Copyright 2019 AIP Publishing.^57^

The ability to model liver functions is paramount in the drug development process as the liver is the main site involved in xenobiotic and drug metabolism in the human body.^35^ Traditional 2D liver cell cultures have been widely adopted by research laboratories as they are cheap, easy to handle, and amenable for high-throughput screening. However, 2D models lack the complexity of the native liver architecture, generating monolayers in which primary cells rapidly lose hepatocyte-related functions.^36^ Thus, 2D liver systems end up being not properly functional in response to drugs and toxins. Animal models, which however can recapitulate what physiologically happens *in vivo*, fail to reconstruct human liver mechanisms due to interspecies discrepancies in the metabolic pathways.^37^ Thus, to overcome these limitations, 3D culture approaches based on human cells are rapidly gaining popularity. In this context, Liver-on-Chip (LoC) platforms have hence been developed by several research groups aiming at improving predictions from physio-pathologically based PK and PD models. Furthermore, LoC platforms capable of recapitulating pivotal features of liver physio-pathology are expected to increase the robustness of compounds screening for hepatic toxicity earlier in the DDP as more than 30% of drug recalls are due to drug-induced liver injury (DILI).^38^ Among all, acetaminophen (APAP) is the most employed compound to assess drug-induced toxicity in newly developed LoC models, and it is one of the most complex compounds that undergoes various pathways of hepatic clearance.^39^ Riahi *et al.*^40^ tested two doses of APAP (5 and 10 *μ*M) on bioprinted HepG2 spheroids cultured in a polydimethylsiloxane (PDMS)-based LoC device to automatically quantify changes in transferrin and albumin biomarkers expression, finding a decreased biomarkers production upon drug administration. By adopting a similar platform, Shin *et al.*^41^ noticed reduced levels of albumin production and an increase in glutathione S-transferase-α (GST-α) from bioprinted primary human hepatocyte (PHH) spheroids embedded in methacrylated gelatin (GelMA) after being treated with APAP. GST-α is a liver biomarker which increases after acute liver injury.^42^ Similar spheroid-like structures of Matrigel™-embedded HepG2 were cultured inside the OrganoPlate™ platform (MIMETAS) in the study conducted by Jang *et al.*^43^ The OrganoPlate is composed of three culture chambers separated by two phaseguides, and it is arranged in a 356-well plate format to increase the experimental throughput [Fig. 1(a)]. To reflect the *in vivo* situation where hepatocytes are exposed to an indirect flow without physical barriers, cell-laden hydrogel constructs were seeded in two lateral chambers, and the medium was perfused along the central channel. Good cell viability, greater albumin and urea productions, and enhanced CYP1A activity were obtained in the 3D perfused system compared to the static condition. To validate the microfluidic device for drug-induced toxicity tests, 25 mM APAP was administered to the HepG2 cells. Such operation decreased hepatic viability down to 10% and 20% for 3D perfused culture and 3D static culture, respectively. This result is in line with previous studies where the presence of fluidic perfusion accentuated drug toxicity effects. Drug sensitivity assessment was also performed by Zhao *et al.*^44^ that treated HepG2 cells co-cultured with immortal human aortic endothelial cell line (HAEC) in a 3D liver lobule-like environment with APAP at increasing doses (i.e., 5, 10, and 20 mM) for 24 h (acute toxicity). HepG2 cells cultured in a 3D environment were more sensitive to APAP-induced toxicity compared to traditional 2D monolayers, whereas, when co-cultured with HAEC in a 3D configuration, HepG2 cells showed higher cellular viability under the same level of APAP treatment. Such outcomes demonstrate the importance of hepatocytes-endothelial cells heterotypic interactions to improve cellular tolerance to APAP-induced toxicity. Weng *et al.*^45^ developed a LoC platform operated by a 24-channel peristaltic pump used to radially direct medium flow from six inlets to mimic the *in vivo* lobular flow from the portal vein to the central vein [Fig. 1(b)]. In this device, primary rat liver cells cultured in a radially symmetric configuration maintained good and stable albumin and urea secretions as well as CYP3A4 activity for two weeks. Moreover, the authors were able to mimic zonal APAP-induced hepatotoxicity, thanks to the perfusion gradient (i.e., oxygen gradient) generated from the six peripheral inlets toward the central outlet. In particular, 1.2 g/l APAP was perfused in the device for 12 h: most of the cellular damage was found in the inner region (zone 2), while the outer region (zone 1) was unaffected, proving the positive effect of the zonation successfully generated in the LoC device.

Oxygen monitoring is a functional and well-established approach to assess drug toxicity on liver cells *in vitro*. For instance, Ehrlich *et al.*,^46^ recently, embedded oxygen microsensors into HepG2/C3A spheroids cultured in disposable PDMS microwells within a perfused LoC device to assess the toxicity levels (i.e., degree of mitochondrial damage) of the drugs valproate (antiepileptic compound) and stavudine (antiretroviral compound), which are known to cause hepatic steatosis in patients. Bavli *et al.*^47^ used a similar system to monitor cellular oxygen uptake after rotenone (it impairs normal functions of the mitochondrial complex I, inducing oxidative stress and apoptosis even at low concentrations)^48^ and troglitazone (it is an antidiabetic and anti-inflammatory drug that causes severe DILI)^49^ delivery in HepG2 cells cultured in their LoC platform [Fig. 1(c)]. Oxygen consumption immediately decreased when cells were exposed to increasing doses of rotenone (1, 50, and 200 *μ*M), eventually reaching 35%, 27%, and 15% of normal respiration upon 12 h treatment, respectively. Similarly, oxygen consumption declined when hepatocytes were treated with increasing doses of troglitazone (350, 500, and 2000 *μ*M), achieving 32%, 15%, and 8% of normal respiration after 24 h exposure, respectively. Thus, both rotenone and troglitazone displayed a dose-dependent cellular damage. Troglitazone was also adopted by Vernetti *et al.*^50^ to demonstrate the predictability of their microfluidic device (sequentially layered, self-assembly construct) encompassing PHH and endothelial cells in a physiologic cell ratio (i.e., 1 endothelial cell:5 PHHs).^51^

*In vivo*, drug administration into the human body takes place by means of the vascular system. Thus, to develop physiologically relevant tissues for drug toxicity and safety testing, the inclusion of engineered models of the vasculature has been considered in recently developed *in vitro* biomimetic systems. Lai *et al.*^52^ adopted a poly(octamethylene maleate (anhydride) citrate) (POMaC) microwell composed of an endothelialized lumen (human umbilical vein endothelial cells, HUVEC) and surrounding parenchymal tissue (HepG2:HUVEC= 9:1). After 7 days of culture, endothelial cells self-assembled into a primitive organization spreading in the parenchymal space (early stage of angiogenesis), adequate to maintain alive and functional 600 *μ*m-thick cellular constructs for the entire culture period. To model systemic drug administration, terfenadine was delivered in the endothelial lumen, and its concentration as well as the levels of its metabolite fexofenadine was measured in both the inner vasculature and the parenchymal space. To the same purpose, by means of a sacrificial bioprinting technique, Massa *et al.*^53^ were able to generate HUVEC vessels inside HepG2/C3A cell-laden GelMA hydrogels. By perfusing 30 mM APAP in the endothelial channels for 48 h, they showed the intrinsic protective role of the endothelial cells toward parenchymal cells as cell mortality was higher at HUVEC site compared to adjacent hepatocytes: probably the presence of the endothelial barrier delayed drug diffusion toward the parenchymal space and/or the HUVECs was able to metabolize the drug preventing hepatocyte injury. Considering liver architecture, Ortega-Ribera *et al.*^54^ developed a sinusoidal-mimicking layered platform to fluidically stimulate liver sinusoidal endothelial cells (LSECs) plated on a polyethylene terephthalate (PET) membrane in the top chamber of the device with a continuous and homogeneous shear stress, whereas PHHs were cultured in the bottom chamber [Fig. 1(d)]. In particular, perfused hepatocyte-LSEC co-cultures were compared to hepatocyte monocultures in terms of albumin production, urea synthesis, and CYP3A4 enzymatic activity. As expected, dynamic co-cultures were able to maintain higher CYP3A4 levels compared to monocultures, probably due to effective paracrine crosstalk between hepatocytes and functional endothelial cells. Moreover, the authors adopted the toxicants troglitazone and tolcapone to study hepatic resistance: concentrations of these drugs previously proposed to be hepatotoxic in other *in vitro* studies did not show hepatotoxicity in their functional model. This means that hepatic resistance of drugs should always be tested in physiologically relevant *in vitro* models as some—potentially promising—drugs that have been withdrawn for liver toxicity after 2D studies might have resulted not dangerous if examined in more physiological conditions. As animal studies are still the gold standard in drug toxicities studies, they are often considered as human relevant models although evident species-related differences exist. In this context, Jang *et al.*^55^ developed a perfused PDMS-based liver-chip with two culture chambers separated by a Extracellular Matrix (ECM)-coated porous membrane. The system, composed of PHH, LSECs, hepatic stellate cells (HSC), and Kupffer macrophages (KC) to mimic liver sinusoid structure, was able to reproduce albumin production level (∼20 to 70 *μ*g/day/10^6^ cells) similar to that estimated *in vivo* (50 *μ*g/day/10^6^ cells) and CYP activities comparable to those exhibited by freshly isolated hepatocytes. The drug JNJ-2 (property of Janssen) was adopted by the authors to show species-related differences of drugs effects. In fact, JNJ-2 is a compound that was previously showed to cause liver toxicity (i.e., fibrosis) in rats, but in the human liver-chip proposed by Jang and colleagues, it showed no toxicity even after 14 day of daily treatment. The lack of a response similar to rat studies in the human liver-chip confirms interspecies differences of drug responses in the liver. Additionally, Methotrexate (MTX) was used as model drug for steatosis: daily administration of MTX at 1, 10, 30 *μ*M for 7 days led to lipid accumulation and stellate cells activation. Hepatotoxicity was evaluated by means of daily administration of the drug bosentan (it causes cholestasis in humans, 1, 3, 10, 30, or 100 *μ*M) which resulted in decreased albumin secretion and demonstrated an *in vivo*-relevant toxic response (C_max_ = 7.4 *μ*M associated with DILI).^56^

Parallel to DILI modeling, several drugs affect the activity of metabolizing enzymes, thus leading to either a reduced or increased toxicity of other drugs when combined. Drug–drug interactions (DDI) were recently modeled by Deng *et al.*^57^ in a liver sinusoid-on-chip platform [Fig. 1(e)]. The composition, proportion, and spatial arrangement of cells were investigated to mimic the physiological features of the *in vivo* sinusoid, with the implementation of two parallel channels for liver artificial blood and biliary efflux with opposite flow directions. The two channels, separated by a 1-*μ*m pore size polycarbonate (PC) membrane, hosted HepG2 embedded-basement membrane extractant and human stellate LX-2 cells seeded on the bottom part of the membrane, and human umbilical vein EAhy926 cells cultured on the top of the membrane. The authors modeled the interaction of 50 *μ*M Rifampicin (RIF) or 25 *μ*M Ciprofloxacin (CPFX) or 50 *μ*M Omeprazole (OME) and APAP. When APAP was mixed with RIF or CPFX, the levels of lactate dehydrogenase (LDH) decreased, indicating lower APAP toxicity when it is combined with RIF or CPFX. Vice versa, when APAP was mixed with OME, LDH levels significantly increased, meaning that this blend enhanced APAP toxicity. The drug RIF was also used by Trietsch *et al.*^58^ as a proof of principle for toxicity assays in a 3D cell culture platform, showing higher mortality levels with increasing doses and exposure times. The aforementioned systems were thus capable to model, predict, and correctly respond to drug toxicity testing, and are all amenable for further and more complex drug studies.

## HEART-ON-CHIP FOR DRUG TESTING

**FIG. 2.**
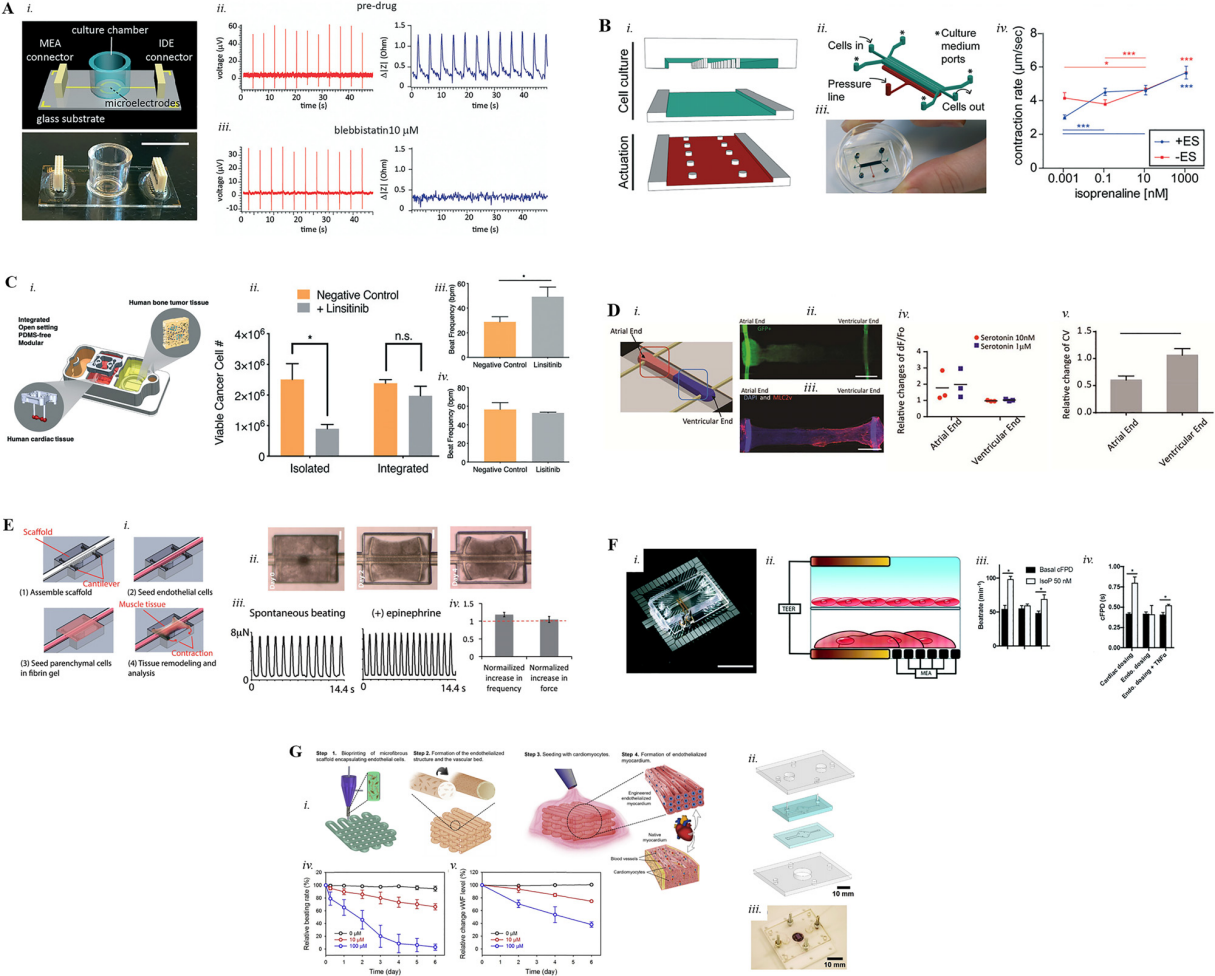
(a) (i) Concept and real picture of the device. Scale bar is 1 cm; platform validation with blebbistatin: hiPSC-CMs field potential (measured by the MEA, red) and contraction (measured by the IDE, blue) before drug treatment (ii), and after 10 *μ*M blebbistatin administration (iii). Republished with permission from Qian *et al.*, Lab Chip **17**, 1732–1739 (2017). Copyright 2017 Royal Society of Chemistry, Clearance Center, Inc.^61^ (b) Heart-on-a-chip device fabricated from three PDMS layers, aligned and irreversibly bonded (i) and (ii); real picture of an actual 3D heart-on-chip device (iii); (iv) contraction rate measured within cardiac constructs in response to increasing isoprenaline concentration (10^−12^–10^−6^ M) in the presence (+ES, blue) or absence (−ES, red) of electrical stimulation (*p <0.05, ***p <0.0001). Republished with permission from Marsano *et al.*, Lab Chip **16**(3), 599–610 (2016). Copyright 2016 Clearance Center, Inc.^70^ (c) Schematic of the system with engineered Ewing sarcoma (ES) tumor and cardiac tissues (i), cultured either with microfluidic perfusion (integrated) or in isolation; viability of ES tumors and cardiac tissues with and without Linsitinib treatment (12 *μ*M, 72 h) comparing isolated and integrated conditions (ii); beat frequency of cardiac tissues after exposure to Linsitinib (12 *μ*M) (mean ± s.e.m., n = 11) in isolation (iii) and in the integrated platform (iv). Republished with permission of Chramiec *et al.*, Lab Chip **20**(23), 4357–4372 (2020). Copyright 2020 Clearance Center, Inc.^77^ (D) (i) Concept of the Biowire II platform with atrio-ventricular ends; (ii) atrial Green Fluorescent Protein (GFP+) and ventricular (GFP−) CMs are placed at the opposite ends of the system suspended between two POMaC wires. Scale bar = 0.5 mm; (iii) ventricular end of the tissues stained positive for myosin light chain 2v (MLC2v). Scale bar = 0.5 mm; (iv) difference in Ca^2+^ transient amplitude after serotonin administration, normalized to the baseline (N = 3, two-way ANOVA with Sidak's test); (v) value of the conduction velocity upon ranolazine application after normalization to the baseline (mean ± SD, N = 4, p = 0.0007, student's t test). Reprinted with permission from Zhao *et al.*, Cell **176**(4), 913–927 (2019). Copyright 2019 Elsevier.^79^ (e) (i) Steps of the cell seeding process in the device; (ii) real images of the cardiac tissue modeled around the scaffold from day 0 to day 4. Scale bar, 200 *μ*m; (iii) changes in cantilever displacements during spontaneous contraction of the construct and after epinephrine (10 *μ*M) stimulation; (iv) quantification of the variations in beating frequency and contraction force under epinephrine stimulation. Reproduced with permission from Lai *et al.*, Adv. Funct. Mater. **27**, 1–11 (2017). Copyright 2017 John Wiley and Sons.^52^ (f) (i) Picture of the TEER–MEA chip. Scale bar is 2 cm; (ii) concept of the platform: endothelial monolayer cultured on top of the PET membrane while cardiomyocytes on top of MEAs, and both cell types cultured between the two sets of TEER electrodes. (ii) Isoproterenol directly administered to the cardiomyocyte channel show an increase in both the beat rate by 80% (iii) and the corrected field potential duration (cFPD) by 90% (iv) whereas it did not have effect when administered through the endothelial channel; isoproterenol administered through the damaged endothelial channel (+TNF-α) shows a significant increase in both the beat rate by 28% (iii) and the cFPD by 42% (iv). Republished with permission from Maoz *et al.*, Lab Chip **17**, 2294–2302 (2017). Copyright 2017 Copyright Clearance Center, Inc.^83^ (g) (i) Steps showing the procedure of fabricating the endothelialized myocardium using the 3D bioprinting method; exploded view of the device: two-layer microfluidic bioreactor sandwiched by a pair of polymethylmethacrylate (PMMA) clamps (ii) and real picture of the bioreactor containing a bioprinted scaffold (iii); relative beating (iv) of the endothelialized myocardial tissues and the levels of vWF expression (v) by the endothelial cells after increasing doxorubicin concentration. Reprinted with permission from Zhang *et al.*, Biomaterials **110**, 45–59, Copyright 2016 Elsevier.^85^

The recent advent of microfluidic technologies and the improvements gained in the field of stem cells led to the development of functional cardiac *in vitro* models amenable for regenerative medicine studies. These systems have the objective of mimicking important morphological and functional features of the cardiac milieu, such as the anisotropic organization of cardiomyocytes (CMs) and the electromechanical stimulation to generate a synchronous contraction beating.^32^ Several 2D and 3D heart models have been developed to enhance and assess the maturation of the reproduced cardiac tissue, its functionality and its response to drugs as well as exogenous substances (e.g., environmental pollutants^59^). An example is the work of Stancescu *et al.*^60^ that considered a 2D cellular model integrated within Biomedical Microelectromechanical Systems (BioMEMS) able to model critical aspects of the *in vivo* myocardial functions such as electrical conduction and contractile force of cardiomyocytes (CMs). In particular, to measure those parameters, CMs derived from human embryonic stem cells (hESC-CMs) were patterned on fibronectin-coated multielectrode arrays (MEA) in order to track the electrical activity of cells as well as on fibronectin-coated silicon cantilever microchips to measure their contractile force. The combination of these two parallel subsystems enabled the investigation of toxicity-related parameters in response to norepinephrine, verapamil, and sotalol administration, and each drug indicated effects in line with clinical data. With the same purpose, Qian *et al.*^61^ seeded human induced pluripotent stem cells derived-CM (hiPSC-CM) monolayers onto a glass substrate with patterned microelectrodes. Such microelectrodes consisted of both MEA and interdigitated electrodes (IDEs) to measure field potential and contraction of CMs, respectively. Blebbistatin, a compound that decreases cardiac contractility, and norepinephrine, a drug that increases cardiac beating rate, were adopted to assess electromechanical alterations in cardiac cells. After incubation with 10 *μ*M blebbistatin, the IDE recording showed a flat baseline signal while the action potential was not affected [Fig. 2(a)]. On the contrary, 400 nM of norepinephrine treatment increased the conduction velocity inside the tissue monolayer by 38%. Similarly, Zhang *et al.*^62^ generated monolayers of primary neonatal rat cardiomyocytes onto an array of sixteen IDEs to assess beating changes after drug administration. Verapamil and Doxorubicin (DOX) were administered to the cultures, and already after 10 min of 125 nM incubation, there was a decrease in contractility, beating rate, and amplitude, known effects of these drugs.^63,64^ In particular, doxorubicin is a drug with well-known cardiotoxicity [0.1 *μ*M inhibiting concentration (IC_50_)], and its primary circulating metabolite is doxorubicinol, known to elicit a powerful cardiotoxic reaction.^65^ Rat cardiomyocytes were also adopted by Lind *et al.*^66^ in a study where a multimaterial 3D printing technique was employed to integrate soft strain gauge sensors within a pattern to provide real-time noninvasive contraction readouts. To recapitulate the anisotropic laminarity of cardiac structures, grooved filaments (60 *μ*m-wide) capable of guiding cardiomyocytes self-assembly were printed on a glass surface. Verapamil was also used here to show negative chronotropic outcomes for spontaneous beating constructs, whereas isoproterenol induced a positive chronotropic effect. In a similar way, Kujala *et al.*^67^ micromolded soft gelatin on standard MEA to engineer laminar cardiac tissues. hiPSC-CMs exhibited an increase in beating rate and a shortening of the QT-interval after incubation with 10 *μ*M isoproterenol in agreement with previous studies.^68^

Even if micropatterned-based systems seem to reproduce the expected drug effects on cardiac cells, there are some concerns about the level of maturation that the cells can achieve, which instead can be provided through 3D cell culture systems. The 3D cell cultures are able to mimic the *in vivo* cardiac microenvironment and enhance the CMs' maturation by coupling the 3D architecture, in which CMs normally live, with mechanical and electrical cues.^69^ These systems involve the formation of cardiac tissue coupled with the presence of a scaffold matrix, typically a ECM-derived material such as fibrin or collagen in which CMs are embedded. An example of functional 3D cardiac tissue is the work of Marsano *et al.*^70^ that investigated the effects of mechanical stimuli in a “beating heart on chip.” This PDMS-based microfluidic platform has been designed to mimic the mechanical stimulation experienced by cells in the native myocardium and to assess the effects of drugs on spontaneously beating or stimulated cardiac cells. The device was realized by the assembly of three layers of PDMS on a glass slide to obtain two microchambers separated by a PDMS membrane. In particular, the cell culture chamber (top compartment) is subdivided by means of two rows of hanging posts into (i) a central channel in which the 3D cell construct is generated by embedding hiPSC-CMs in a fibrin gel, and (ii) two side channels for medium replenish. The actuation compartment (lower compartment), once pressurized, has the function to bend the membrane between the two compartments, generating a compression in the 3D cell construct (i.e., 10%–15% uniaxial stretch). Compared to nonpaced tissues, cyclic mechanical stimulation was found to improve not only the architecture, maturation, and functionality of the 3D microtissues, but it also showed a positive chronotropic effect on isoprenaline (1 nM) treatment, a drug commonly used to treat bradycardia [Fig. 2(b)]. The same system was recently improved, adding needle microelectrodes to either stimulate cardiac microtissues (CMT)^71^ or record field potentials generated during the heartbeats, allowing to assess electrophysiological alterations due to administered drugs.^72^ Isoprenaline was also recently adopted as model drug in an integrated heart-on-a-chip platform developed by Zhang *et al.*^73^ The PDMS-based device hosts hiPSC-CMs cultured on gelatin hydrogels and allows for continuous stimulation and real-time monitoring, thanks to the inclusion of platinum wire electrodes and gold electrode arrays. In particular, the authors tested the effects of sequential administration of verapamil (0.5 mM) and isoprenaline (1 *μ*M) on both stimulated and nonstimulated cardiac constructs. Verapamil treatment provoked a beating frequency decrease in both conditions, whereas the subsequent isoprenaline addition was able to restore the beating rate only in the stimulated group, showing a more physiological behavior of the electrically stimulated constructs. In this view, Ronaldson-Bouchard *et al.*^74^ developed a platform to demonstrate that an electrical pacing of increasing intensity can enhance the differentiation and functional maturation of fetal hiPSC-CMs into an adult phenotype. 6 mm-long and 1.8 mm-diameter constructs were generated and stretched between two flexible PDMS pillars and subjected to electrical stimulation using carbon rods. After the administration of 1 *μ*M isoproterenol, they detected a positive chronotropic response with half-maximum effective concentration (EC_50_) values matching the ones of clinical trials.^75^ Cardiac tissue maturation is thus an important prerequisite to obtain physiological responses to drugs. The displacement of bending PDMS pillars to measure cardiac tension was also adopted by Truitt *et al.*^76^ who studied the cardiac *in vitro* afterload by means of cardiac microtissues (CMT) composed of iPSC-CMs (93%) and human mesenchymal stem cells (huMSC, 7%) generated between the pillars. In particular, CMT were cultured between both soft and stiff PDMS pillars for 5 days before being treated with 1 *μ*mol/l Sunitinib, a drug used to treat solid tumors that cause hypertension. The authors showed that human CMT cultured on stiff pillars exhibited higher cardiotoxicity upon sunitinib administration compared to CMT cultured on soft pillars. This result shows that increased *in vitro* afterload (i.e., stiff PDMS pillars) enhances sunitinib-induced toxicity in human CMT. Similarly, in a recent study conducted by Chramiec *et al.*,^77^ a PDMS-free platform to study antitumor efficacy and cardiac safety of newly synthetized drugs was fabricated. The platform itself is a stable polysulfone structure where the cardiac tissue, composed of 75% hiPSC-CMs and 25% normal human dermal fibroblasts encapsulated in a fibrin gel, is generated between two elastic polyoxymethylene pillars, which is capable of inducing tissue elongation and alignment. Linsitinib (12 *μ*M, clinically used dosage), a novel anticancer drug used to suppress tumor growth that failed during phase II clinical trial, was employed to treat primary Ewing Sarcoma (ES) tumor cells and cardiac cells in isolated cultures when integrated in a fluidically connected platform. In isolation cultures, the drug was able to induce tumor death and enhance beating frequency of cardiac cells, not matching clinical data, whereas no response was observed in the integrated platform, in line with clinical results [Fig. 2(c)].^78^ This study signifies the necessity of developing more predictive experimental systems that can be employed to better predict clinical outcomes at earlier stages in development, avoiding time-consuming and expensive late-stage drug failures.

As the aforementioned study, many research groups are moving toward the development of PDMS-free systems to diminish or eliminate hydrophobic compound binding. For example, Zhao *et al.*^79^ developed the Biowire II platform which enables the growth of cardiac tissues suspended between two parallel flexible POMaC wires glued along two opposite ends of a polystyrene microwell. The generated tissue is composed of both atrial and ventricular CMs embedded in a collagen-Matrigel mixture patterned at the two opposite ends of the microwells with a mixture of the two cell types in the transition zone. To validate their model, the authors adopted serotonin and ranolazine toxicants which are reported to have preferential atrial effects.^80^ As expected, at the atrial ends of the platform, serotonin treatment caused Ca^2+^ transients to increase, whereas ranolazine caused conduction velocity reduction; ventricular ends were not affected by either of drugs [Fig. 2(d)]. Such system is thus interesting to study drugs with either complex or not well understood mechanisms of action. With a similar platform, named I-Wire, Sidorov *et al.*^81^ engineered an elongated 3D cardiac tissue construct of 300–400 *μ*m in diameter between two titanium wires. Isoproterenol (1 *μ*M) was used to demonstrate a significant increase in the generated force and contraction velocity, whereas 6 *μ*M blebbistatin was adopted to inhibit contractility and assess the effect on stiffness of the construct, which decreased by 23%. Dogbone-shaped cardiac constructs of hiPSC-CMs were also previously adopted by Huebsch *et al.*,^82^ which, compared to hiPSC-CM 2D monolayers, showed a prolonged chronotropic effect upon isoproterenol treatment and onefold higher inhibiting concentration (IC_50_) for verapamil, disclosing a clinically relevant drug responsiveness.

To recapitulate drug ADME process, new generation microfluidic systems should incorporate a functional endothelial barrier to mimic drug vascular administration and diffusion toward target and nontarget tissues. The majority of complex OoC do not consider endothelial modules, and compounds are often directly delivered to cells. To simulate an endothelialized environment, Lai *et al.*^52^ developed a microfluidic system composed of PDMS microwells, where 3D cardiac microtissues could generate around a vascular lumen and two T-shaped microcantilevers. In particular, the cantilevers were embedded with carbon electrodes to monitor the forces generated by the constrained cardiac tissues. The continuous administration of epinephrine (10 *μ*M) within the luminal vasculature showed an instant growth in both the contraction frequency and spontaneous beating of cardiac constructs, whereas no significant increase was noticed in tissue contraction force [Fig. 2(e)]. In another study, Maoz *et al.*^83^ adopted human umbilical vein endothelial cells (HUVECs) and hiPSC-CM monolayers to develop an endothelialized myocardium within their heart-on-a-chip platform. In particular, monolayers of endothelial and cardiac cells were cultured within two PDMS chambers separated by a porous (0.4 *μ*m pore size) PET membrane. By means of transepithelial electrical resistance (TEER) electrodes and MEAs, they were able to quantify the integrity of the endothelial barrier and to monitor electrical activity of cardiomyocytes, respectively. In particular, electrodes were positioned close to the CM monolayer to allow both excitation and field potential recording of CMs (MEAs) as well as measurement of the electrical impedance to evaluate the integrity of the endothelium (TEER) [Fig. 2(f)]. For cardiotoxicity studies, they assessed the effects of continuous isoproterenol (50 nM) administration into either intact or damaged vascular channels within the platform: in the first case, no effect on cardiomyocytes monolayers was detected, whereas in the second case, cardiac beating rate increased by 28%, which is in line with clinical outcomes.^84^ In this context, Zhang *et al.*^85^ developed an endothelialized myocardium-on-a-chip for cardiovascular toxicity evaluation with bioprinted scaffolds. In particular, a GelMA-alginate laden HUVEC bioink was used to generate scaffolds with biomimetic anisotropic patterns, which was then cellularized with hiPSC-CMs. The authors performed a dose-dependent study by means of the anticancer drug doxorubicin that induced a decrease in the beating rate of cardiomyocytes from 94.5% for the control tissues to 66.0% and 2.78% (near 0 bpm) for CMs treated with 10 and 100 *μ*M doxorubicin, respectively. Similarly, also the levels of von Willebrand factor (VWF, secreted by endothelial cells) decreased from >90% for the controls to 76.0% and 35.3% for tissues dosed with doxorubicin at 10 and 100 μM, respectively [Fig. 2(g)]. In recent times, Weng *et al.*^86^ developed a PDMS three-chamber microfluidic device separated by 30 *μ*m spaced PDMS structures to simultaneously and rapidly analyze the anticancer and potential cardiotoxicity of chemotherapeutics. The device hosted a monolayer of human induced pluripotent stem cell derived endothelial cells (hiPSC-ECs) in the central channel, fibrin-laden hiPSC-CMs in one lateral channel and colon adenocarcinoma cell line SW620 spheroids in the other lateral channel. The system was challenged by perfusing in the endothelial channel doxorubicin (IC_50_ = 0.1 *μ*M) and oxaliplatin, a drug without known cardiac side effects (IC_50_ = 4.2 *μ*M). Doxorubicin reduced the growth of cancer cells and decreased the spontaneous beating rate and conduction velocity close to anticancer IC_50_. Vice versa, oxaliplatin reduced the growth of cancer cells and reached similar cardiac functions only when exceeding (>33 *μ*M) IC_50_ in accordance with *in vivo* outputs. Thus, a well-developed endothelial monolayer can recapitulate the barrier functionality of endothelial cells and should be taken into consideration when approaching new pharmacokinetic/pharmacodynamic (PK/PD) studies. Furthermore, the aforementioned studies provide functional and efficient models for probing drug-induced cardiovascular toxicity and should be considered and adopted in preclinical trials to reduce late-stage drug withdrawals.

## LIVER–HEART ON CHIP FOR DRUG SAFETY TESTING

**FIG. 3.**
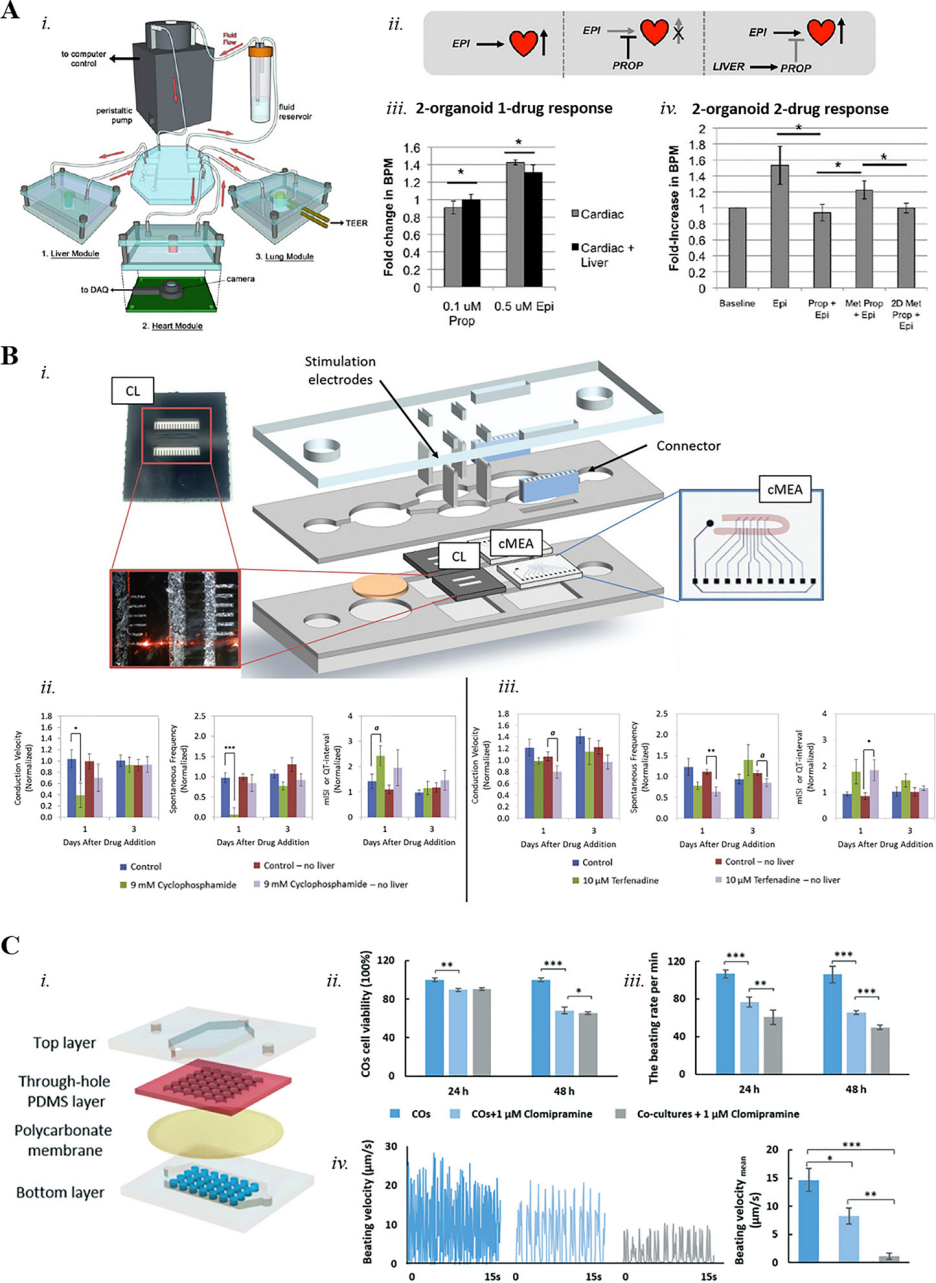
(a) (i) Concept of the modular multitissue chip hardware system setup for culture of three different models connected via a central fluid routing breadboard; (ii) illustration of the two drug, two organoid interactions; (iii) cardiac organoids beating values in response to both 0.1 *μ*M propranolol and 0.5 *μ*M epinephrine with presence and absence of the liver module; (iv) increases in beating from epinephrine addition are blocked by 0.1 *μ*M propranolol in the absence or presence of liver module (both organoids or 2D hepatocytes). 2D hepatocytes metabolism is reduced in comparison to hepatic organoids. Statistical significance: *p <0.05. Reproduced with permission from Skardal *et al.*, Sci. Rep. **7**, 8837 (2017). Copyright 2017 Authors, licensed under a Creative Commons Attribution (CC BY) license.^88^ (b) (i) Schematic of the pumpless microfluidic system hosting cardiac and liver co-culture and the interface used to measure functionality (cardiomyocytes on cantilevers and on MEA chips); (ii) heart–liver co-culture and cardiac response upon cyclophosphamide treatment (9 mM) in presence and absence of hepatocytes. All values are normalized to the control before drug administration (a p <0.08, *p <0.05, ***p <0.001); (iii) heart–liver co-culture and cardiac response upon terfenadine treatment (10 *μ*M) in presence and absence of hepatocytes. All values are normalized to the control before drug administration (a p <0.08, *p <0.05, **p <0.01). Reprinted with permission from Oleaga *et al.*, Biomaterials **182**, 176–190 (2018). Copyright 2018 Elsevier.^91^ (c) (i) Exploded view of the liver–heart organoids-on-chip system: top layer, through-hole PDMS layer, polycarbonate porous membrane and bottom layer. Assessment of clomipramine-induced cardiotoxicity (1 *μ*M clomipramine for 24 and 48 h) after liver metabolism on the liver–heart organoids-on-chip: (ii) cell viability and (iii) beating rate of cardiac tissues with and without liver organoids. N = 3, mean ± SD (*P <0.05, **P <0.01, ***P <0.001); (iv) beating motion track of cardiac organoids with different drug treatments and the quantification of mean beating velocity of each condition (N = 3, mean ± SD; *P <0.05, **P <0.01, ***P <0.001). Republished with permission from Yin *et al.*, Lab Chip **21**, 571–581 (2021). Copyright 2021 Clearance Center, Inc.^95^

Lately, many research groups focused their attention on the development of *in vitro* platforms able to simultaneously culture multiple organ models. These systems are of great relevance in the drug screening and safety fields as they are potentially capable to mimic *in vitro* the ADME process of drugs as it occurs in the human body.^2^ As previously stated, the liver is the organ mainly involved in compound metabolism, whereas most off-target toxicities influence the heart.^11,32,87^ Therefore, a great interest has arisen in conceiving liver–heart models that can model and predict off-target cardiac toxicity upon liver metabolism of newly developed and recalled drugs. A few platforms have been created with the aim of integrating hepatic and cardiac tissues into one single device for predictive drug testing applications. Among these Multi-Organs-on-Chip (MOoC) platforms, the *Ex vivo* Console of Human Organoids (ECHO) developed by Skardal *et al.*^88^ is a perfusion-driven and modular microfluidic platform designed to provide physiological responses to toxic agents and pharmaceuticals. In the ECHO, spherical organoids of both cardiac (i.e., hiPSC-CMs) and liver cells (i.e., PHH, HSC, KC) were bioprinted in separated reactors and then fluidically connected through a circulatory perfusion system (i.e., plug and play system). Once the long-term viability and functionality of liver and cardiac organoids was proved through immunostaining of specific-tissues biomarkers (i.e., albumin, α-GST and creatine kinase), the effects of epinephrine (0.5 *μ*M) and propranolol (0.1 *μ*M) on the beating rate of cardiac organoids were evaluated within the platform [Fig. 3(a)]. Epinephrine normally induces an increase in beating rate of cardiac cells whereas propranolol has the opposite effect. The authors assessed the effects of the two compounds independently, first on the system encompassing only cardiac cells (i.e., cardiac-only system) and then on the system when both cardiac and liver cells were cultured (i.e., dual-organoid system). Administration of propranolol (0.1 *μ*M) caused a slight (10%) decrease in the beating rate when considering only cardiac cells in the platform, while epinephrine (0.5 *μ*M) increased (40%) the beating rate. In the dual-organoid system, propranolol and epinephrine were delivered in the upstream liver module before reaching the cardiac site. In this condition, the liver was able to mitigate drug effects as propranolol treatment did not decrease the cardiac beating rate, which was instead increased (30%) by epinephrine. Subsequently, the authors assessed the effect of a combination of the two compounds on the dual-organoid system. By delivering propranolol first and epinephrine immediately afterwards, the authors demonstrated that most of the administered propranolol was metabolically inactivated by the liver organoids since the final result was a significant increase (25%) in beating rates induced by epinephrine. These studies demonstrate the importance of liver metabolism in studying drug effects on the heart when dealing with drug safety applications.

A different MOoC platform used to study cardiotoxicity induced by drugs, and their metabolites was used by Oleaga *et al.*^89–91^ and by McAleer *et al.*,^92^ consisting in a pumpless gravity driven system encompassing different organ modules. The device is composed of two outer polymethylmethacrylate (PMMA) sheets and two-to-five inner PDMS gaskets that define the fluidic pathway between communicating compartments. PHHs were cultured on a collagen-coated glass coverslip, whereas hiPSC-CMs were cultured on silicon cantilevers to record mechanical activity (i.e., contractile force) and on custom MEA (cMEA) chips to measure electrical activity (i.e., conduction velocity, beat frequency, and QT-interval). The platform was validated with an acute dose-response study of two different drugs, namely, cyclophosphamide (9 mM) and terfenadine (10 *μ*M), due to their known toxicity alterations upon hepatic metabolism. Specifically, Cyclophosphamide is a noncardiotoxic parent drug that generates a cardiotoxic metabolite (i.e., acrolein) after liver metabolism, whereas terfenadine is a cardiotoxic parent drug that generates a noncardiotoxic metabolite (i.e., fexofenadine) following the metabolic process within the liver. After cyclophosphamide administration in the liver compartment, after 24 h the platform was able to detect the cardiotoxicity of acrolein which significantly affected cardiac electrical activity (i.e., reduced conduction velocity, beat frequency and prolongation of QT-interval). Moreover, the transformation of terfenadine into fexofenadine had a protective effect from terfenadine original cardiotoxicity, with no significant cardiac functional change [Fig. 3(b)]. Within the same platform, the effects of Doxorubicin (DOX), Atorvastatin (ATR), Valproic acid (VPA), APAP, and N-Acetyl-m-aminophenol (AMAP) were assessed on both cardiac and liver cells.^89^ Following literature data, DOX (5 *μ*M) showed both hepatotoxic and cardiotoxic side effects, whereas ATR (100 *μ*M) and VPA (2 mM) had both cardioprotective and hepatotoxic effects. The authors also showed that while APAP (5 mM) has a known hepatotoxic effect, it did not show cardiotoxicity in the platform, whereas AMAP (5 mM), which is the commonly used nonhepatotoxic control of APAP, induced mild cardiotoxicity. Such outcomes show the ability of MOoC systems to identify potentially dangerous effects on target and off-target tissues and thus the necessity of their employment as valuable *in vitro* tools in everyday research studies. Recently, Pires de Mello *et al.*^93^ added a synthetic skin surrogate at the latter device to mimic drug topical absorption. The skin was modeled by means of a Strat-M membrane (Millipore), a synthetic membrane on top of the culture chambers. After administration of diclofenac sodium, ketoconazole, hydrocortisone, and APAP, the authors showed that in most cases the topical administration did not impair hepatic and cardiac functions compared to the systemic one, and 24 h after absorption, the concentration of the topically dosed drugs was relatively low, validating the potency of barrier properties. Another example of MOoC is the model developed by Zhang *et al.*,^94^ consisting of an automated modular device hosting two microbioreactors for the culture of liver and cardiac organoids. The bioreactor possesses two hemi-chambers of PDMS for cell culture hosted in two PMMA supports. Liver organoids were constructed with PHHs, whereas cardiac organoids were constructed with iPSC-CMs. Such cultures were fluidically connected via a microfluidic-controlling breadboard for timed routing of fluids and were monitored in real-time by oxygen sensors and immunosensors to check the amounts of oxygen concentration and generated biomarkers, respectively. To validate the model, they adopted the drug capecitabine, a prodrug that can undergo enzymatic activation by hepatocytes to the active form 5-fluorouracil (5-FU), exerting cardiotoxic effects. Liver and heart organoids were also implemented in a PDMS platform recently developed by Yin *et al.*^95^ Specifically, the device is composed of two chambers separated by a 0.4 *μ*m-pores membrane. The upper chamber hosts 3D hiPSC-derived hepatocytes organoids, whereas the bottom chamber consists of an array of micropillars for 3D culture of hiPSC-CM organoids [Fig. 3(c)]. To assess drug-related toxicity, clomipramine was administered in the liver chamber of the system. Clomipramine is an antidepressant drug which is metabolized into desmethylclomipramine (active form) by CYP2D6 of hepatocytes.^96^ The study showed that clomipramine at relevant clinical concentrations (i.e., 1 *μ*M)^97^ is sufficient to lower Ca^2+^ influx and increase cardiotoxicity in heart organoids upon liver metabolism. Another example of heart–liver microfluidic device to recapitulate the side effects (i.e., cardiotoxicity) of an anticancer drug *in vitro* is the one developed by Kamei *et al.*^98^ The platform is a PDMS-based microfluidic device fabricated with on-chip integration of pneumatic valves and peristaltic micropumps establishing a closed circulation system. It is composed of two layers: an upper perfusion layer hosting two culture chambers and a control layer that contains both valves and micropumps. By culturing HepG2 cells and human primary cardiomyocytes in the closed loop system, the authors demonstrated that cardiotoxicity was mainly due to doxorubicinol, principal metabolite obtained from hepatic metabolism of doxorubicin.^65^

As previously stated, liver and heart toxicities are the principal cause of drug candidate failures and recalls during the DDP. To develop efficient OoC models for drug safety studies, it is thus paramount to use device materials amenable for such purpose. PDMS is the most widely adopted materials as it is easy to handle, inexpensive, transparent, permeable to oxygen, and biocompatible.^31^ However, PDMS absorbs drug compounds, especially those that are highly lipophilic, which can be deleterious in dose-dependent drug studies.^99^ To overcome this limitation, Skardal *et al.*^100^ recently developed a PMMA-based MOoC platform able to host up to six organoid systems (i.e., liver, heart, lung, vascular, testis, brain or colon). The device itself is composed of four layers: a lid layer, a chamber layer (PMMA sheet), a microfluidics layer, and a porous membrane layer. Liver organoids were generated from 80% PHHs, 10% HSCs, and 10% KCs, whereas cardiac organoids were composed of 90% iPSC-CMs and 10% human primary cardiac fibroblasts. Organoids were first exposed to recalled drugs in order to perform single toxicity testing on isolated liver and heart systems. In particular, bromfenac, tienilic acid, and troglitazone caused liver cell damage (i.e., reduced viability). Similarly, astemizole, cisapride, mibefradil, pergolide, rofecoxib, terodiline, and valdecoxib caused cardiotoxicity as well as changes in the cardiac beat kinetics of heart cells. However, *in vivo* there are several organ–organ interactions that are mimicked *in vitro* by the reciprocal responses of liver and cardiac organoids in the microfluidic system. To model this physiological condition, the authors administered the chemotherapeutic drugs capecitabine (20 *μ*M) and cyclophosphamide (20 *μ*M) in the liver compartment of the MOoC system and evaluated cardiotoxic outcomes. Upon capecitabine treatment, systems without liver organoids did not show significant toxicity in any downstream organoids, whereas systems containing liver organoids were able to biotransform capecitabine into 5-FU, and toxicity was observed in both cardiac and lung organoids. Likewise, when cyclophosphamide was delivered in the platform, systems without liver organoids did not metabolize the prodrug and thus no toxicity was observed, whereas with liver organoids presence, metabolism of the compound into a toxic metabolite (i.e., acrolein) was toxic for both the cardiac and lung constructs. This is a further evidence of systemic toxicity dependency on liver metabolism.

## OUTLOOK AND CONCLUSIONS

In the drug development process (DDP), the majority of current methods to assess drug safety in pre-clinical phases are often costly and inefficient. Indeed, pre-clinical trials mainly rely on simplistic two-dimensional *in vitro* models or animal experimentation.^8^ Organs-on-Chip (OoC) and microfluidic technologies propose fascinating engineered tools to generate *in vitro* human organ models that might be adopted to investigate in a more characteristic way both drug toxicity and safety of newly developed and recalled compounds in agreement with the 3Rs principles (Replacement, Reduction, and Refinement, Directive 2010/63/EU). In particular, liver and heart toxicities account for the 90% of drug withdrawal from the market,^101^ and, thus, microphysiological systems encompassing these two organs have been developed and widely adopted in basic research. In fact, microphysiological systems can recapitulate the physiological structure of human organs with enhanced tissue functionalities to study drugs effects *in vitro*. The use of these systems has showed beneficial effects in terms of drugs prediction, and their exploitation in the early phases of the DDP is expected to dramatically contribute to reduce costs, time, and ethical concerns related to animal experimentation. Moreover, such *in vitro* models can speed up the DDP itself by shortening the gap that usually arises between pre-clinical and clinical phases.^17^ Nonetheless, the path to market for OoC devices is still long, and it mildly depends on the capability to combine more than one organ into a single platform, fundamental to examine drug-related PK/PD profiles. In fact, OoC encompassing only one organ that are not able to mimic and predict drugs' systemic effects followed hepatic biotransformation as they occur *in vivo*. This impairs a throughout study of the effects of promising molecules which undergo liver metabolism as well as molecules that once metabolized can cause unpredicted systemic toxicity (i.e., cardiotoxicity). In this scenario, the integration of interconnected liver and cardiac functional models in *in vitro* systems holds the promise to outperform traditional assays through specific safety tests on both parent drugs and their metabolites, minimizing the occurrence of false positive/negative results and eventually enriching the DDP process.^89^ In this review, we presented liver-on-chip and heart-on-chip models currently used to study hepatotoxicity and cardiotoxicity, respectively. Moreover, we conferred an overview of the recently developed integrated MOoC platforms, comprising interconnected liver and heart models, capable to study and detect *in vitro* cardiotoxicity of drugs upon hepatic metabolism. Furthermore, most MOoC platforms have been designed to embed biosensors (e.g., MEA),^102^ making them particularly suitable for real-time screening of physio-pathological characteristics (e.g., cardiomyocytes beating properties). However, it is worth underlining that the vast majority of these platforms still rely on two-dimensional cultures. In this review, we presented the few recent MOoC platforms encompassing 3D organ systems, which were developed to provide a better representation of the *in vivo* microenvironment. Nevertheless, substantial improvements are still required for both technical and biological standpoints. For example, MOoC devices could be particularly interesting in studying cancer and immunotherapies. The ability to integrate tumor tissues together with other organs in one single platform and study their mutual interactions may offer enriched knowledge on cancer development and progression as well as off-target systemic toxicity. For instance, therapies based on immune checkpoint inhibitors might be tested on MOoC platforms owing to their expected ability to predict pharmacokinetics and thus provide more comprehensive responses regarding off-target complications in both liver and heart. Additionally, as MOoC platforms enable the use of cells of human origin within *in vitro* platforms where physio-pathologic interactions can be studied and validated, they will speed up the DDP, eventually reducing the risks of post-marketed drug withdrawals. Considering human cells, it is well known that primary liver cells are challenging to maintain in *in vitro* cultures due to their scarce availability and difficult logistics.^36,103^ PHHs represent the gold standard as they can maintain high and stable hepatic functions similar to freshly isolated cells,^104^ but their scarce availability and short lifespan limit their use mainly to short-term studies.^105^ The recent advent of hiPSC-derived hepatocytes could allow their use for long-term studies as they were shown to exhibit specific hepatocyte functions, including albumin secretion and CYP450 expression, and can provide an unlimited supply of cells.^106,107^ However, with the available differentiation protocols, it is hard to generate hiPSC-derived hepatocytes more closely resembling mature phenotype,^107^ and this limits their usage at the microscale. Despite this, hiPSC hold the potential of becoming the “new gold standard” in OoC as soon as reliable differentiation and culture protocols will be available. Furthermore, hiPSC represent a nearly unlimited source of either healthy or disease-specific patient-derived cells, thus making them ideal to promote precision-medicine models for the DDP.^108,109^ In this view, MOoC systems encompassing hiPSC might be fundamental to model patient-specific differences in response to drug metabolism. Another important consideration must be introduced on the topic of drug–drug interactions (DDI), which is caused when the mechanisms of action of one drug are transformed by another one.^110^ Many new medications are developed every year, and additional interactions among drugs are progressively announced. For instance, in the context of DDI-related cardiotoxicity, the cardiotoxic drug cisapride^111^ is inactivated to safe compounds by liver metabolism when administered alone in humans. However, when co-administered with CYP3A4 inhibitors (e.g., the antifungal ketoconazole), the inactivation of cisapride is impaired, and this indeed caused its withdrawal from the market.^112^ Lee-Montiel *et al.*^113^ were recently able to recapitulate this condition in a hiPSC-based MOoC system where liver and heart individual OoC were fluidically connected. In this view, MOoC models encompassing the liver tissue might have the ability to predict unforeseen medical complications due to DDIs, giving unprecedented information in the DDP, by studying and determining which DDIs effects (e.g., unexpected cardiotoxicity) are due to liver metabolism on other tissues. This can help in determining the correct multidrug therapy for selected patients and thus avoiding the risk of unknown toxic effects due to drug–drug coadministration. In fact, unexpected toxicity due to coadministration may be evidenced just in some patients or classes of individuals that share similar liver/heart characteristics. Personalized MOoC systems might be thus fundamental in clustering both patients and drugs to improve the DDP. Additionally, such systems will be pivotal in the recapitulation of drug ADME process as well as in the study of cancer biology and immune diseases, decreasing and hopefully replacing animal models in line with the 3Rs principles. Considering all this, it is expected that from now on MOoC platforms will progressively move from academic research to Pharma and Biotech industries, with the ultimate ambition of being pivotal in personalized medicine studies.

## Data Availability

The data that support the findings of this study are available from AIP Publishing, John Wiley and Sons, PNAS, Royal Society of Chemistry, Elsevier, and Scientific Reports. Restrictions apply to the availability of these data, which were used under license for this study. Data are available from the authors upon reasonable request and with the permission of AIP Publishing, John Wiley and Sons, PNAS, Royal Society of Chemistry, Elsevier, and Scientific Reports.
